# Improving ECG Interpretation Skills Among Healthcare Providers at Aswan University Hospitals: A Clinical Audit

**DOI:** 10.7759/cureus.102510

**Published:** 2026-01-28

**Authors:** Eiman Yassir Musa Hussain, Razan Mutasim Mahgoub Idris, Reem Ahmed Mohammed Diab, Ahmed Mahmoud Sidahmed Abdullah, Ziyad Tarig Hashim Gabir, Khalid Abdelhadi Ahmed Elbalal, Tarig Osman Mohamedali Ahmed, Randa Mahamoud Hamid Hassan, Othman Yousuf Ibrahim Elhaj, Rana Noureldaim Mustafa Ahmed, Rawaa Ahmed Widaa Ahmed, Shazly Bghdadi Ali Ahmed

**Affiliations:** 1 Pulmonary Medicine, Aswan University Hospitals, Aswan, EGY

**Keywords:** : clinical audit, ecg, interpretation, junior doctor, quality improvement project (qip)

## Abstract

Background: Electrocardiogram (ECG) interpretation is a fundamental skill in cardiovascular diagnostics, crucial for identifying various cardiac conditions and guiding appropriate clinical decisions. Misinterpretation can lead to adverse patient outcomes. This clinical audit aimed to assess and improve the commonest life-threatening cases that can be faced during daily practice at Aswan University Hospitals, Aswan, Egypt.

Methods: This clinical audit involved 124 participants in the second cycle, including house officers, medical officers, and registrars, from Aswan University Hospitals. Data were collected using a pre-made questionnaire and analyzed. A six-stage approach to ECG interpretation was used as the standard for assessment, covering electrical activity presence, ventricular rate, QRS rhythm regularity, QRS width, atrial activity presence, and the relationship between atrial and ventricular activity. A first audit cycle assessed baseline knowledge (N=102), followed by an intervention (training and educational materials based on the new local standards), and then a second audit cycle (N=124) to measure improvement. Ethical approval was obtained from the hospital's institutional review board and ethics committee.

Results: Significant improvements were observed in ECG interpretation skills between the first and second audit cycles. The ability to identify electrical activity increased from 67 (65.7%) to 118 (95.2%), a 29.5% improvement. Knowledge of calculating heart rate improved by 37.0%, from 47 (46.1%) to 103 (83.1%). Recognition of regular heart rate improved from 66 (64.7%) to 100 (80.6%), a 15.9% improvement. Identifying changes in QRS width showed the largest improvement of 40.2%, from 33 (32.4%) to 90 (72.6%). The ability to identify atrial activity increased by 6.7%, from 68 (66.7%) to 91 (73.4%). Finally, recognizing the relationship between atrial and ventricular activity improved by 27.8%, from 49 (48.0%) to 94 (75.8%).

Conclusion: This clinical audit demonstrated a substantial improvement in ECG interpretation skills among healthcare providers at Aswan University Hospitals after the intervention. This reflects enhanced adherence of staff to guidelines, thus optimizing patient care. Continued re-auditing is recommended to ensure sustained implementation of these improved standards.

## Introduction

Electrocardiogram (ECG) interpretation proficiency remains suboptimal among medical trainees globally. Studies from Poland and Saudi Arabia reveal that while students can identify basic ECG elements, they often fail to recognize life-threatening abnormalities due to insufficient training [1]. Al Mousa et al. found that Saudi interns struggled with ECG interpretation despite advanced life support training, reinforcing the need for case-based education [2]. In the UK, only 9.7% of junior doctors reported confidence in ECG interpretation, with no improvement across training levels, suggesting limitations in traditional teaching methods [3]. Online learning approaches, particularly interactive webinars, have shown promise in improving confidence and skill acquisition [4,5]. The objective of this audit was to evaluate baseline ECG interpretation performance among junior doctors against predefined standards based on the six-stage ECG interpretation model. These standards required complete and accurate application of all six interpretation components, followed by implementation of a targeted educational intervention and re-audit to assess compliance and improvement. Collectively, these findings highlight the urgent need for structured, practical, and continuous ECG education to enhance diagnostic accuracy and patient care.

## Materials and methods

Study design and setting

This was a prospective clinical audit conducted at Aswan University Hospitals over the period 15/01/24 to 15/06/24.

Study population and sample size

The audit population included house officers (also referred to as interns), medical officers, and registrars (also referred to as residents) working at Aswan University Hospitals who were routinely involved in ECG interpretation during clinical duties. Participants were recruited using convenience sampling.

A total of 102 physicians participated in the baseline audit cycle, and 124 physicians participated in the re-audit cycle. The predominance of house officers reflects the local clinical practice, where junior doctors are primarily responsible for initial ECG interpretation, particularly during on-call shifts.

Data collection tools and analysis

Data were collected using a structured questionnaire developed to assess ECG interpretation skills according to the six-stage interpretation model (see Appendix). The questionnaire content was reviewed by senior clinicians with experience in cardiology and acute care to ensure content relevance and clarity.

Responses were collected and analyzed. Results were expressed as frequencies and percentages, with comparisons made between baseline and re-audit cycles to assess changes in performance. No inferential statistical testing was performed.

ECG interpretation standards

Assessment of ECG interpretation skills was based on a six-stage structured approach, with predefined standards requiring complete and accurate application of all six interpretation components. These standards were formulated by hospital consultants to align with local clinical practice and expectations (Table 1).

**Table 1:** Structured Six-Stage Protocol for ECG Interpretation

Stage	Description
1	Is there any electrical activity?
2	What is the ventricular (QRS) rate?
3	Is the QRS rhythm regular or irregular?
4	Is the QRS width normal (narrow) or broad?
5	Is atrial activity present?
6	How is atrial activity related to ventricular activity?

Audit cycle

The clinical audit was conducted in three progressive phases:

Phase I - Baseline Assessment (First Cycle)

The initial assessment was carried out among 102 physicians to evaluate their existing competency in ECG interpretation. Using the six-stage protocol, responses were examined for accuracy, completeness, and pattern recognition. This phase helped identify core gaps in interpretation skills across clinical ranks.

Responses were assessed for completeness and correctness across all six interpretation stages, and overall compliance with the predefined standards was recorded.

Phase II - Interventions

The intervention phase involved a multifaceted educational strategy designed to enhance participants’ ECG interpretation skills. This included didactic training sessions systematically addressing each of the six ECG interpretation stages, ensuring a structured understanding of the diagnostic approach. Tailored guideline summaries and visually engaging reference posters were disseminated to reinforce key concepts and provide quick-reference tools during clinical decision-making. Structured interpretation templates were also distributed to guide consistent documentation practices. Additionally, interactive discussions and Q&A rounds created dynamic learning environments that allowed participants to clarify uncertainties and deepen their comprehension. Collectively, these interventions aimed to standardize interpretation methods and promote diagnostic consistency across all levels of clinical responsibility.

The intervention was delivered over a four-week period by senior physicians with experience in ECG interpretation and medical education. Training sessions were conducted in small groups to facilitate interaction and practical application.

Phase III - Post-intervention Reassessment (Second Cycle)

A reassessment was conducted with 124 physicians to evaluate the effectiveness of the interventions. The same questionnaire was administered, and a comparative analysis was performed to measure improvements in interpretation accuracy. Emphasis was placed on both qualitative shifts in reasoning and quantitative increases in compliance with standard criteria.

Ethical Considerations

The audit was conducted in accordance with ethical guidelines, ensuring data confidentiality and patient privacy. Approval was obtained from the hospital’s institutional review board (IRB) and ethics committee prior to commencement (approval number: 10021024). 

## Results

A total of 226 participants were assessed across the two audit cycles; the samples in the first and second cycles were independent and not paired, as different participants were included in each cycle using a structured, locally formulated six-question framework designed by hospital consultants. This approach enabled a progressive and measurable assessment of core competencies essential for accurate ECG analysis. Notably, there was significant overall improvement between the first and second audit cycles across all parameters.

In the initial stage, determining whether any electrical activity was present, accuracy rose from 67 (65.7%) to 118 (95.2%), reflecting a 29.5% improvement. Similarly, identification of the ventricular (QRS) rate improved markedly, from 47 (46.1%) to 103 (83.1%), yielding a 37.0% gain. The ability to recognize the regularity of the QRS rhythm increased more modestly, from 66 (64.7%) to 100 (80.6%), a 15.9% improvement.

The most significant relative gain was observed in the classification of QRS width, which improved by 40.2%, from only 33 (32.4%) to 90 (72.6%). Recognition of atrial activity exhibited a more modest increase of 6.7%, from 68 (66.7%) to 91 (73.4%). Meanwhile, understanding the relationship between atrial and ventricular activity improved from 49 (48.0%) to 94 (75.8%), indicating a 27.8% gain.

Table 2 summarizes these improvements across various key aspects of ECG analysis. Overall, the comparison between the first and second cycles indicates a consistent and substantial enhancement in the participants' ECG interpretation capabilities across all assessed domains.

**Table 2:** Comparison of ECG Interpretation Skills Between First and Second Audit Cycles

ECG Interpretation Skill	First Cycle; N (%)	Second Cycle; N (%)	Improvement (%)
Is there any electrical activity?	67 (65.7%)	118 (95.2%)	29.50%
What is the ventricular (QRS) rate?	47 (46.1%)	103 (83.1%)	37.00%
Is the QRS rhythm regular or irregular?	66 (64.7%)	100 (80.6%)	15.90%
Is the QRS width normal (narrow) or broad?	33 (32.4%)	90 (72.6%)	40.20%
Is atrial activity present?	68 (66.7%)	91 (73.4%)	6.70%
How is atrial activity related to ventricular activity?	49 (48.0%)	94 (75.8%)	27.80%

To ensure a representative understanding of documentation practices across the clinical workforce, a total of 226 participants were assessed during the two audit cycles. For participant distribution across roles, the composition remained consistent across both cycles, with interns forming the majority at 206 (91.2%), followed by medical officers at 18 (8.0%), and residents contributing 2 (0.9%) (Table 3). This distribution underscores the pivotal role of interns in shaping documentation habits, while also highlighting the need to engage medical officers and residents in quality improvement interventions to ensure standardized compliance across all tiers of healthcare providers.

**Table 3:** Distribution of Healthcare Participants by Role

Role	N (%)	N (%)
Interns	206	91.20%
Medical officers	18	8.00%
Residents	2	0.90%
Total	226	100%

## Discussion

The findings of this clinical audit demonstrate a clear improvement in ECG interpretation performance following the implementation of a targeted educational intervention based on a structured six-stage interpretation model. This supports existing evidence that focused, systematic training can enhance ECG interpretation skills among junior doctors [6].

Improvement was most pronounced in core interpretative domains such as ventricular rate assessment and recognition of QRS width abnormalities [7,8]. These components are frequently encountered in daily clinical practice and were repeatedly emphasized during the intervention through posters, structured templates, and case-based discussions [9,10]. The observed pattern suggests that repetitive exposure and reinforcement of foundational steps are particularly effective in improving interpretation accuracy. In contrast, more complex interpretative elements showed comparatively smaller gains, likely reflecting the need for longer-term training and continued clinical exposure.

The audit’s findings are consistent with previous studies reporting suboptimal ECG interpretation skills among interns and junior physicians, particularly when training is unstructured or inconsistent. Prior literature has demonstrated that case-based and structured educational approaches are more effective than traditional lecture-based teaching in improving ECG competency, which aligns with the improvements observed following the intervention in this audit [11-15].

Interpretation of the results should consider several important limitations. The participant population differed between audit cycles and consisted predominantly of interns, reflecting local staffing patterns where junior doctors are primarily responsible for initial ECG interpretation. While this supports the relevance of the audit to real-world clinical practice, it also limits generalizability and introduces potential confounding when comparing baseline and re-audit results. In addition, the audit assessed departmental-level performance rather than individual paired outcomes, and formal psychometric validation of the questionnaire was not performed.

Despite these limitations, the audit successfully identified gaps in ECG interpretation practice, implemented a feasible educational intervention, and demonstrated measurable improvement against predefined standards. The findings support the role of regular audit and feedback cycles in improving clinical practice and highlight the value of structured ECG interpretation frameworks in junior doctor education.

## Conclusions

This clinical audit effectively demonstrated a significant improvement in the ECG interpretation skills of healthcare providers at Aswan University Hospitals. The substantial gains across various aspects of ECG analysis reflect a successful intervention and adherence to clinical guidelines. These improvements are anticipated to translate into optimized patient care and improved outcomes in the management of various health conditions. Continued re-auditing is strongly recommended to ensure the sustained implementation of these enhanced standards and foster a culture of continuous learning and quality improvement. 

## Figures and Tables

**Figure 1:**
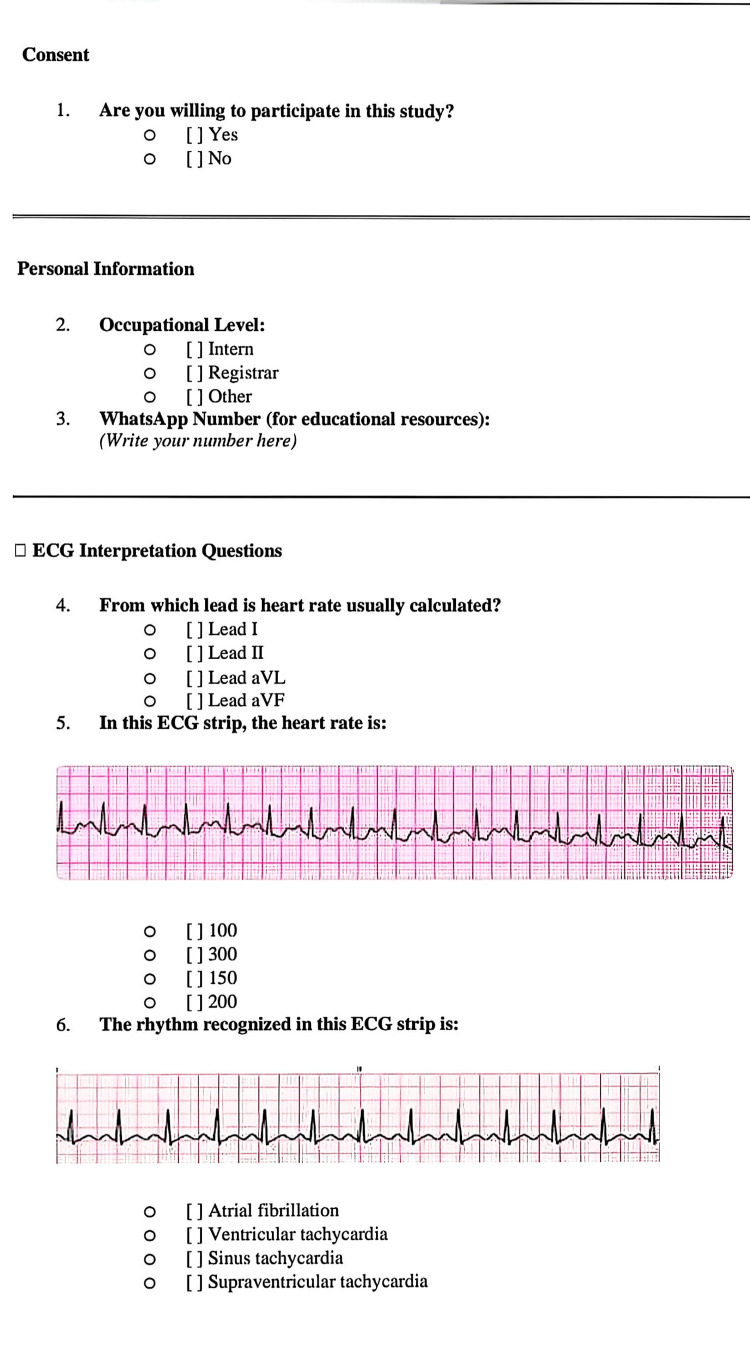
ECG Interpretation Questionnaire (First Page) NOTE: The audit population included house officers (also referred to as interns), medical officers, and registrars (also referred to as residents) working at Aswan University Hospitals who were routinely involved in ECG interpretation during clinical duties. The term “medical officers” refers to a broader non-training clinical category and was included under the “other” option in the questionnaire.

**Figure 2:**
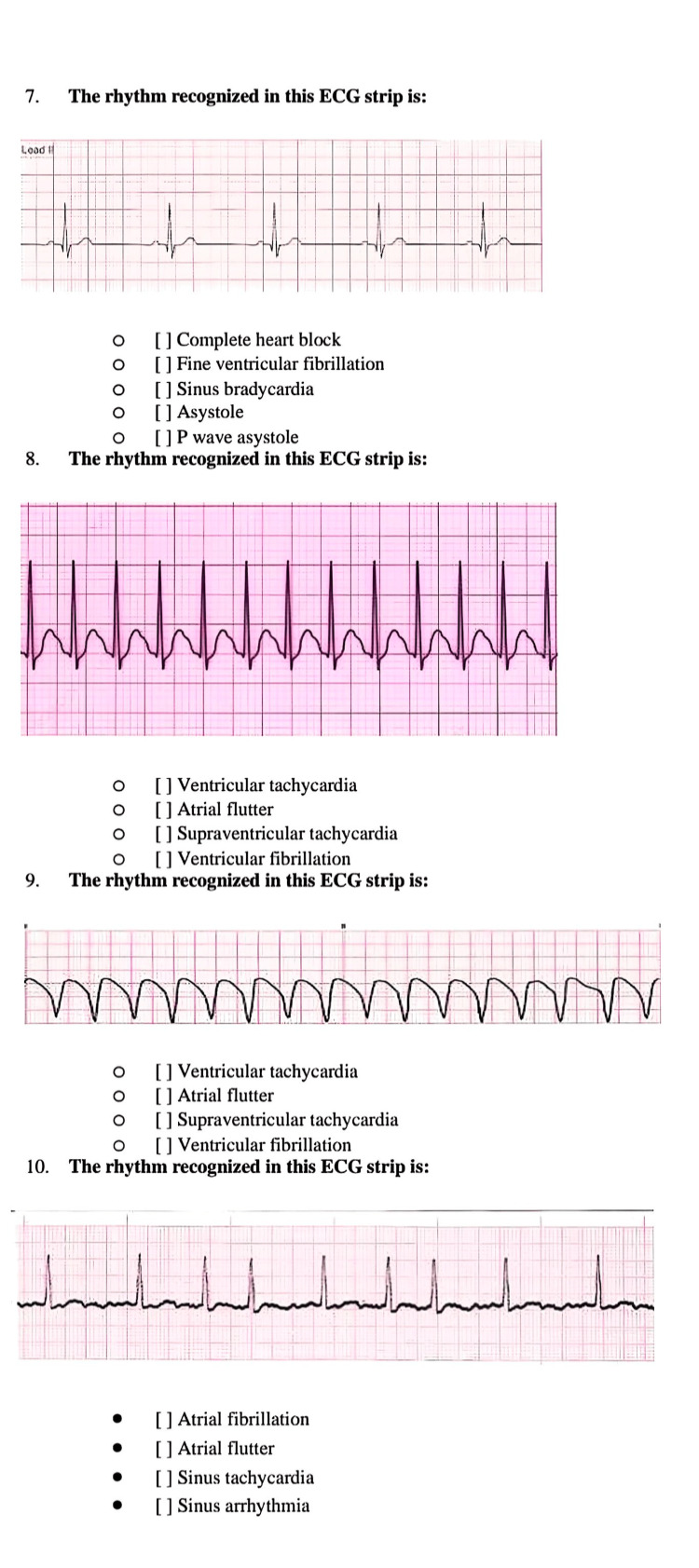
ECG Interpretation Questionnaire (Second Page)

**Figure 3:**
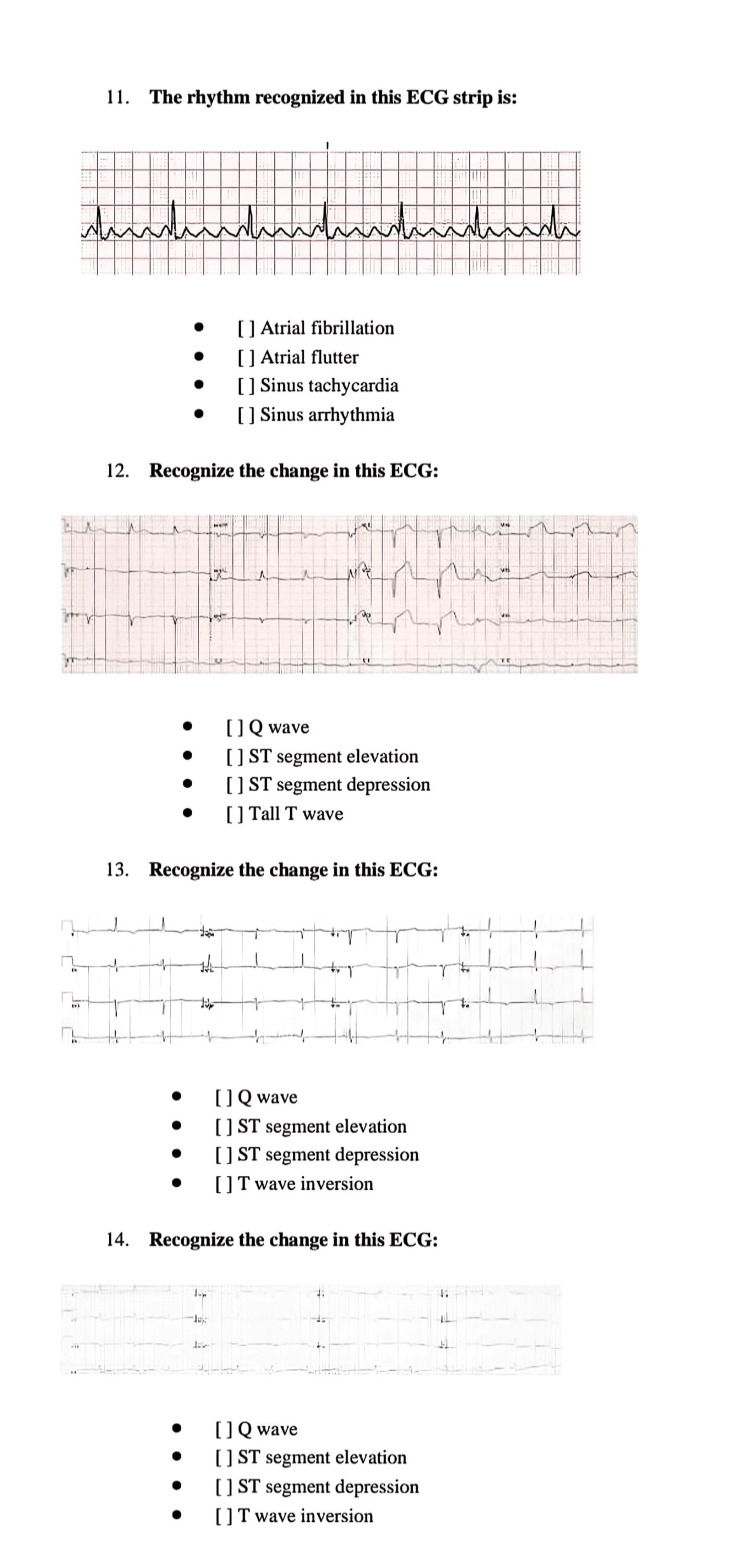
ECG Interpretation Questionnaire (Third Page) Standardized questionnaire used to assess clinical proficiency in electrocardiogram interpretation. The tool was reviewed for content validity and implemented across participating sites to support audit and educational objectives.

## Appendices

Figures  1-3 show the questionnaire distributed to participants for the clinical audit. 
